# Identification and Structural Characterization of Interneurons of the *Drosophila* Brain by Monoclonal Antibodies of the Würzburg Hybridoma Library

**DOI:** 10.1371/journal.pone.0075420

**Published:** 2013-09-17

**Authors:** Beatriz Blanco Redondo, Melanie Bunz, Partho Halder, Madhumala K. Sadanandappa, Barbara Mühlbauer, Felix Erwin, Alois Hofbauer, Veronica Rodrigues, K. VijayRaghavan, Mani Ramaswami, Dirk Rieger, Christian Wegener, Charlotte Förster, Erich Buchner

**Affiliations:** 1 Institute of Clinical Neurobiology, University of Würzburg, Würzburg, Germany; 2 Department of Neurobiology and Genetics, Theodor-Boveri-Institute, Biocenter, University of Würzburg, Würzburg, Germany; 3 Institute of Zoology, University of Regensburg, Regensburg, Germany; 4 National Centre for Biological Sciences, Tata Institute of Fundamental Research, Bangalore, India; 5 School of Genetics and Microbiology and School of Natural Sciences, Smurfit Institute of Genetics and Trinity College Institute of Neuroscience, Trinity College Dublin, Dublin, Ireland; University of Houston, United States of America

## Abstract

Several novel synaptic proteins have been identified by monoclonal antibodies (mAbs) of the Würzburg hybridoma library generated against homogenized *Drosophila* brains, e.g. cysteine string protein, synapse-associated protein of 47 kDa, and Bruchpilot. However, at present no routine technique exists to identify the antigens of mAbs of our library that label only a small number of cells in the brain. Yet these antibodies can be used to reproducibly label and thereby identify these cells by immunohistochemical staining. Here we describe the staining patterns in the *Drosophila* brain for ten mAbs of the Würzburg hybridoma library. Besides revealing the neuroanatomical structure and distribution of ten different sets of cells we compare the staining patterns with those of antibodies against known antigens and GFP expression patterns driven by selected Gal4 lines employing regulatory sequences of neuronal genes. We present examples where our antibodies apparently stain the same cells in different Gal4 lines suggesting that the corresponding regulatory sequences can be exploited by the split-Gal4 technique for transgene expression exclusively in these cells. The detection of Gal4 expression in cells labeled by mAbs may also help in the identification of the antigens recognized by the antibodies which then in addition to their value for neuroanatomy will represent important tools for the characterization of the antigens. Implications and future strategies for the identification of the antigens are discussed.

## Introduction

The concept of identifiable neurons represents a unique feature of invertebrate neuroscience. Neuron identity in general is a consequence of differential cell-specific and temporal regulation of gene expression or other epigenetic mechanisms throughout development. Thus in principle it should be possible to label each individual neuron. Groups of neurons that express a common gene can be detected by localizing the corresponding mRNA by in-situ hybridization or they can be labelled by enhancer trap or gene trap techniques. Alternatively, antibodies can be used to label groups of neurons that contain a common gene product or a common metabolic product of cell-specific gene expression, like e.g. a certain neurotransmitter or a particular post-translational modification (PTM). Thus it is not surprising that antibodies against numerous known proteins, their metabolic products, or their PTMs are available. However, since a large percentage of human genes and their homologues in genetic model systems like *Drosophila* are still uncharacterized, an attractive alternative approach to the identification of cell-specifically expressed genes – and thereby characterization of those cells – is the generation of libraries of monoclonal antibodies against whole tissue homogenates. We have used this approach that originally was developed for *Drosophila* by the group of Seymour Benzer [1], [2], and obtained antibodies against antigens located in neuronal compartments like the cell body, the axon, or the synaptic neuropil, and antibodies that selectively stained individual cells or cell types in the retina or in the brain [3]. In some cases it has been possible to identify the proteins recognized by the antibodies, like cysteine string protein (CSP), synapse-associated protein of 47 kDa (SAP47), Bruchpilot (BRP), Epidermal Growth Factor Receptor Substrate 15 (EPS15), Pigment Dispersing Factor (PDF) precursor (reviewed in [4]). However, this approach “from antibody to gene” has worked so far for antibodies that recognize relatively abundant proteins like those found in all synapses, or, as in the case of the PDF precursor, by a candidate approach when the cells identified by the antibody were already known to contain a certain protein or peptide. Here we present a collection of mAbs that can be used to reproducibly identify small subsets of brain neurons and thus represent valuable tools for cellular neuroanatomy. These antibodies may attain particular importance when the same cells are independently labelled by antisera against known antigens, tissue in-situ hybridization, or by GFP expression in enhancer trap or gene trap lines. Recently, the number of lines expressing Gal4 in subsets of *Drosophila* brain neurons has been significantly increased by high-throughput approaches employing fragments of regulatory sequences of genes expressed in the nervous system [5], [6] (see also ViennaTiles http://stockcenter.vdrc.at). Whenever Gal4 expression and staining by our mAbs overlap it may become possible to identify even rare cell-specific antigens and the genes responsible for their expression. In cases where a mAb identifies the same Gal4 expressing cells in different lines with otherwise non-overlapping expression patterns, the corresponding regulatory sequences can be used for a split-Gal4 approach [7] to transgene expression exclusively in these cells. Here we present the labelling patterns of ten mAbs and examples of co-expression of the unknown mAb antigens and GFP driven by Gal4 lines.

## Materials and Methods

### Fly strains

Canton S (CS) wild type was used throughout the experiments except when stated otherwise. The line 386Y contains a Gal4 enhancer trap P-element in the amontillado gene [8]. The stocks of the Janelia Gal4 collection used here were obtained from the Bloomington stock center and are listed in table 1 (5 digit identifier).

**Table 1:** List of mAbs compared with GFP (detected by anti-GFP (1∶1000) driven by Gal4-lines) or stainings with antibodies against known antigens (pigment dispersing hormone, PDH (1∶1500); myoinhibitory peptide, MIP (1∶1000); neuropeptide F, NPF (1∶300); period protein, PER (1∶2000); serotonin, 5HT (1∶400)).

mAb/subclone	Dilution	Isotype	Gal4-line or antibody [Bloomington stock #]	co-localization + = complete (+) = in subset
ab47/7	(1∶5–1∶20)	IgM κ	R49D04 [38679]	(+)
“	(1∶5–1∶20)	“	R51C07 [38773]	–
“	(1∶5–1∶20)	“	R67E08 [39445]	–
“	(1∶5–1∶20)	“	R57A02 [39878]	(+)
“	(1∶5–1∶20)	“	R75F06 [39901]	–
“	(1∶5–1∶20)	“	R78E11 [40001]	–
“	(1∶5–1∶20)	“	R40E08 [41238]	–
“	(1∶5–1∶20)	“	R42H01 [48150]	–
“	(1∶5–1∶20)	“	R12G09 [48525]	–
“	(1∶5–1∶20)	“	R49C04 [50415]	(+)
ab135/4	(1∶20)	IgM	386Y (amon-Gal4)	–
ab158	(1∶20)	IgM	386Y (amon-Gal4)	(+)
ca8/3/3	(1∶20)	IgM κ	386Y (amon-Gal4)	(+)
“	(1∶20)	“	R59E08 [39219]	–
“	(1∶20)	“	R83H07 [40371]	–
“	(∶20)	“	R14C06 [48604]	–
fb20/1	(1∶5–1∶20)	IgM κ	R49D04 [38679]	–
“	(1∶5–1∶20)	“	R51C07 [38773]	(+)
“	(1∶5–1∶20)	“	R67E08 [39445]	(+)
“	(1∶5–1∶20)	“	R57A02 [39878]	–
“	(1∶5–1∶20)	“	R75F06 [39901]	–
“	(1∶5–1∶20)	“	R78E11 [40001]	–
“	(1∶5–1∶20)	“	R40E08 [41238]	–
“	(1∶5–1∶20)	“	R42H01 [48150]	–
“	(1∶5–1∶20)	“	R12G09 [48525]	–
“	(1∶5–1∶20)	“	R49C04 [50415]	–
nb139	(1∶2)	n.d.	anti-PDH	–
nb168	(1∶20)	IgG	anti-PDH	+
nb169/2	(1∶20)	IgG1	386Y (amon-Gal4)	(+)
“	(1∶20)	“	anti-PDH	–
“	(1∶20)	“	anti-5HT	–
“	(1∶20)	“	R33G11 [45964]	(+)
“	(11∶20)	“	R49C04 [50415]	(+)
nc24	(1∶5)	IgM	anti-PDH	–
“	(1∶5)	“	anti-MIP	–
“	(1∶5)	“	R49D04 [38679]	(+)
“	(1∶5)	“	R51C07 [38773]	–
“	(1∶5)	“	R67E08 [39445]	–
“	(1∶5)	“	R57A02 [39878]	(+)
“	(1∶5)	“	R75F06 [39901]	–
“	(1∶5)	“	R78E11 [40001]	–
“	(1∶5)	“	R40E08 [41238]	(+)
“	(1∶5)	“	R12G09 [48525]	–
“	(1∶5)	“	R49C04 [50415]	(+)
nc53	(1∶5)	IgM	anti-PDH	–
“	(1∶5)	“	anti-NPF	–
“	(1∶5)	“	anti-PER	–

### Generation and characterization of monoclonal antibodies

Hybridoma cell culture, monoclonal antibody production, and isotyping have recently been described [9].

### Wholemount confocal fluorescence microscopy

**Figure 1:**
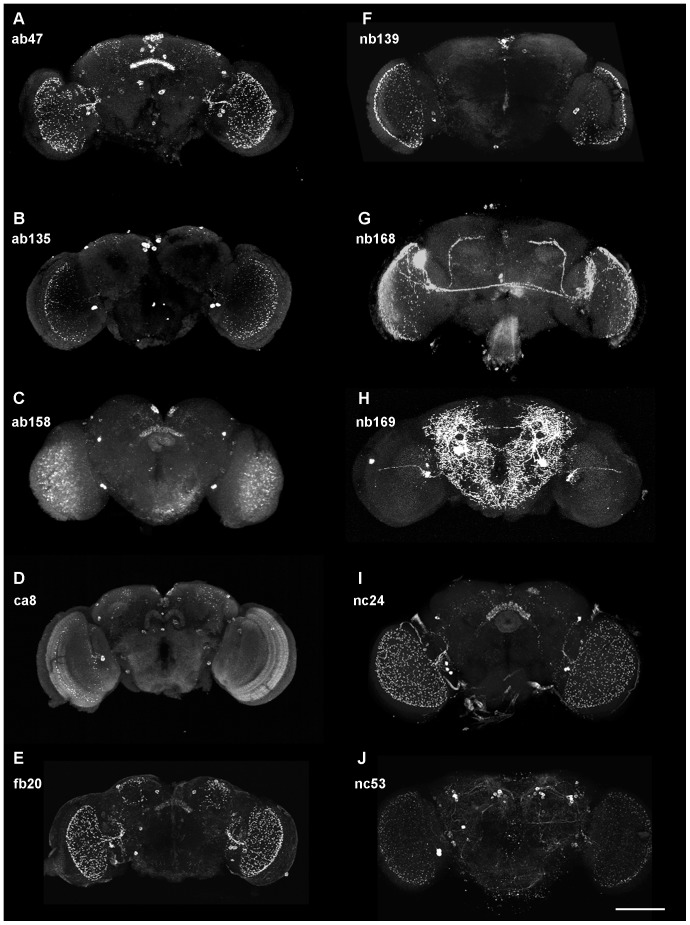
Z-projections of confocal stacks of whole-mount adult *Drosophila* brains (frontal view) stained with ten different monoclonal antibodies of the Würzburg hybridoma library. Note in (H) that the bright spot at the left rim of the projection is due to a staining artefact from outside the brain. Scale bar 100 µm.

**Figure 2:**
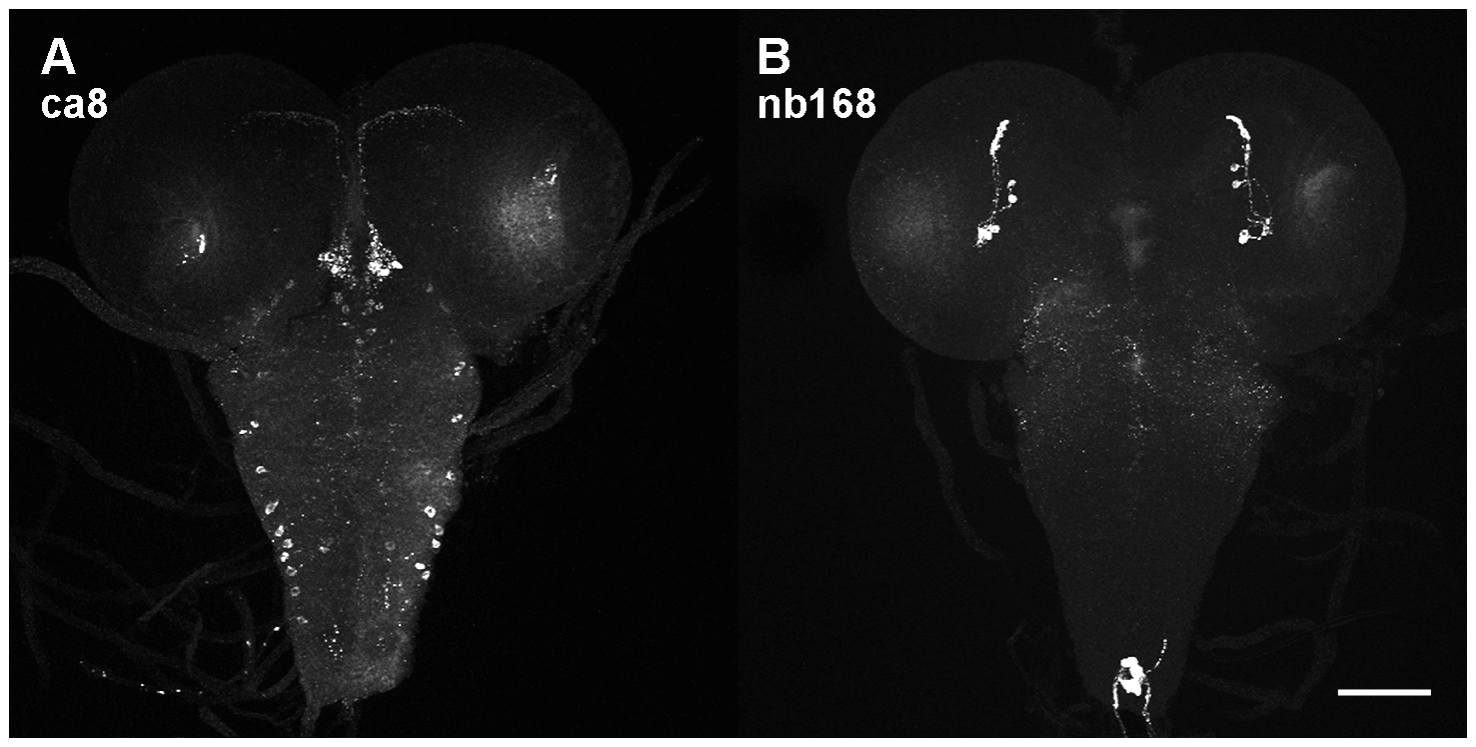
Z-projections of confocal stacks of larval nervous systems stained with two of the monoclonal antibodies shown in Figure 1. Larval staining of nb169 is shown in Figure 4K-M, the other seven antibodies did not produce reliable staining in the larval nervous system. Scale bar 100 µm.

**Figure 3:**
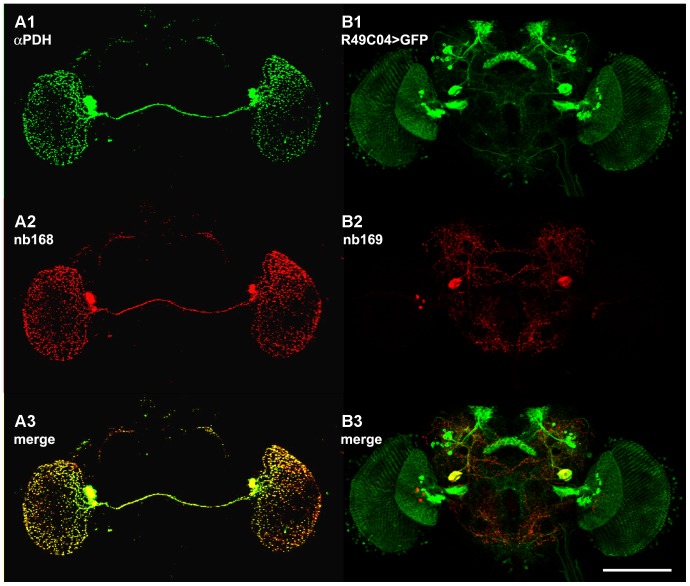
Z-projections of confocal stacks of whole-mount brains double-stained with mAb nb168 and anti-PDH antiserum (A1-3) or mAb nb169 and anti-GFP in a fly expressing GFP driven by Janelia Gal4 line R49C04 (B1-3). The perfect co-localization in A3 indicates that nb168 could recognize *Drosophila* PDF or its precursor protein, whereas co-localization in B3 is restricted to the two very large cells stained by nb169 in the posterior protocerebrum. Scale bar 100 µm.

**Figure 4:**
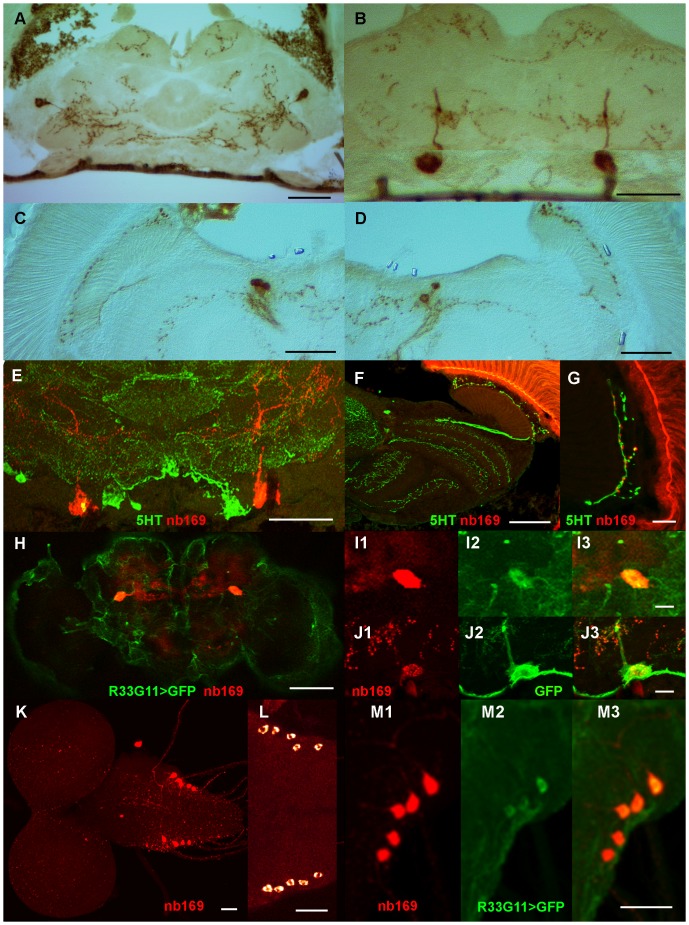
Staining details for mAb nb169. (A-D) The horizontal cryo-sections stained with mAb nb169 and DAB development show the cell bodies in the lateral protocerebrum at the dorso-ventral level of the fan-shaped and ellipsoid bodies (A), the giant neurons in the caudal cellular cortex with their main neurite (in an adjacent section) at the level of the noduli (B), and the cell bodies innervating the accessory medulla, a horizontal layer of the medulla, and a fiber extending to and branching with varicosities in the lamina cortex (C, D). (E-G) Horizontal cryo-sections double-stained with nb169 (red) and anti-serotonin antiserum (green) demonstrate that serotonergic neurons and cells that contain the nb169 antigen are distinct but that their neurites in the lamina cortex are closely apposed. (H-J) GFP (green) expressed under the control of the Janelia Gal4 line R33G11 co-localizes with the nb169 antigen (red) in frontal confocal optical section (H, I1-3) and in horizontal cryo-section (J1-3). (K-M) In the larval nervous system the nb169 antigen (red) is localized in 10 cells (K, L) of which at least 8 also express GFP (green) under the control of R33G11-Gal4 (M, enlargement of K). Scale bars in A-F, H, K-M: 50 µm; in G, I, J: 10 µm.

**Figure 5:**
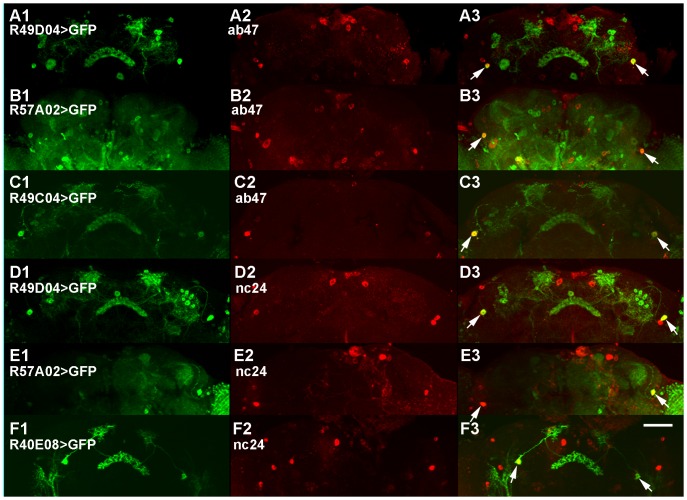
Examples for the co-localization of staining by mAbs ab47 (A-C) and nc24 (D-F) and GFP driven by selected Janelia Gal4 lines. Due to the variability of cell body positions in different individuals it is only suggestive that the Gal4 expression patterns overlap. Scale bar 100 µm.

**Figure 6:**
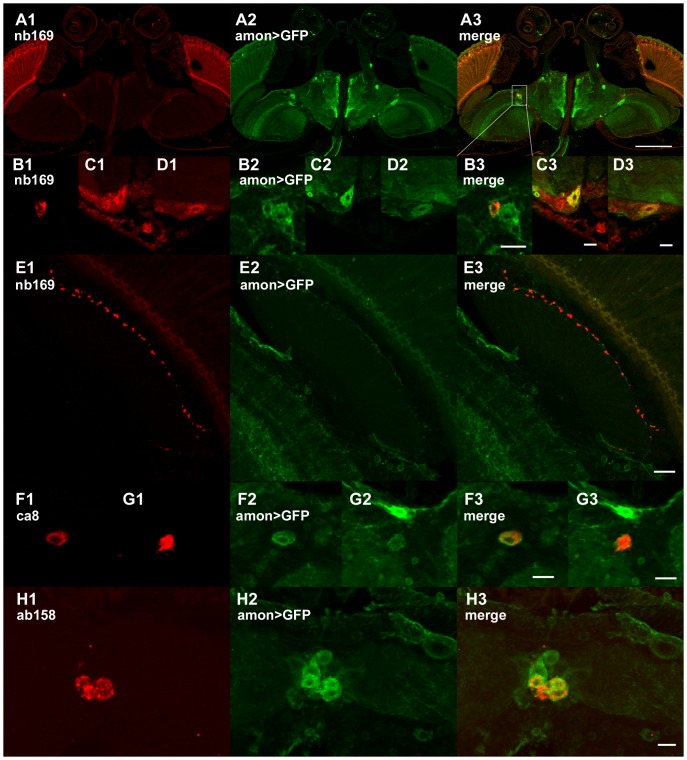
The antigens detected by mAbs nb169, ca8, and ab158 may be related to neuropeptides. (A-E) The cell stained by nb169 (red) (A, enlarged in B), and the giant cells in the caudal protocerebrum (C and D from different sections) all are positive for GFP (green) expressed under the control of 386Y amon-Gal4 which has been shown to selectively mark neuropeptide-containing cells. (E) The neurite of a 386Y *amon*-Gal4>GFP-positive cell at the boundary between lamina cortex and neuropil apparently contains in discrete varicosities the nb169 antigen. (F,G) Examples are shown for co-localization of 386Y *amon*-Gal4-driven GFP and ca8 antigen in cells of the lateral protocerebrum. (H) Cells containing ab158 antigen in the pars intercerebralis, a region containing many neurosecretory cells, are also positive for amon-Gal4-driven GFP. Scale bar in A: 50 µm; in B-H: 10 µm.

Different authors contributing to the stainings in Figures 1–6 used slightly varying procedures for antibody staining of adult or larval whole-mount brains of *Drosophila*. BM (Figure 1A, B, D E, H) followed the detailed description given in [10]. The procedure used by MB and FE (Figure 1F, I, J) has been described in [11], [12] and was followed with minor modifications: Brains were removed from the head capsule in PBS containing 0.5% Triton X-100 (PBS-0.5T) at room temperature (RT) and fixed for 1 h in 4% paraformaldehyde (PFA) in PBS-0.1T. They were washed five times in PBS for 10 minutes each, blocked in PBS-0.5T and 5% normal goat serum (NGS) for 3 h at RT or overnight at 4°C. The brains were incubated for 36 h at 4°C with the primary antibodies diluted as indicated in Table 1 in PBS-0.5T with 5% NGS, washed five times 10 min in PBS-0.5T at RT and incubated overnight at 4°C with two of the following secondary antibodies diluted in PBS with 5% Normal Goat Serum and 0.5% Triton X-100: Alexa Flour 488 goat anti-rabbit IgG (1∶200), Alexa Flour 488 goat anti-mouse IgG (1∶200), Alexa Flour 555 goat anti- rabbit IgG (1∶200), Alexa Flour 555 goat anti-mouse IgG (1∶200) (all from Life Technologies). Finally, the brains were washed 5×10 min in PBS-0.5T at RT and once in PBS-0.1T and mounted in Vectashield™ (Vector Laboratories) for fluorescence microscopy. The brains were examined by confocal microscopy (Leica TSC SPE All pictures were taken at 20×. BBR and MKS (Figures 1C, G, 2, 3, 4H-M, 5, 6F-H) followed the procedure described in [13] with some modifications: Brains of adults or wandering third instar larvae were dissected in PBS, fixed for 30 min – 1 h in 4% PFA in 0.04 M phosphate buffer, washed 3×5 min in PBS-0.3T, blocked at RT for 2 h in 5% NGS in PBS-0.3T, incubated in primary antibody (including chicken anti-GFP 1∶1000) for 48–60 h on a horizontal shaker at 4°C, followed by 6×20 min washes in PBS-0.3T, incubation in secondary antibody as above at dilutions 1∶400 (or Alexa Fluor 488 goat anti-chicken 1∶400, Alexa Flour 568 goat anti-mouse IgG 1∶400) for 12–24 h on a horizontal shaker at 4°C, 6×20 min washes in PBS-0.3T at RT and mounting in Vectashield™. The brains were imaged using confocal microscope Olympus FV1000-IX81 (Tokyo, Japan). Staining patterns clearly similar to the ones shown in Figure 1 were obtained with: ab47 five times in seven stained brains (5/7), ab135 (3/4), ab158 (4/5), ca8 (12/14), fb20 (4/4), nb139 (10/11), nb168 (3/3), nb169 (7/7), nc24 (9/11), nc53 (12/14).

### Cryosections

The procedure for immunostaining of cryosections has recently been described [9]. Dilutions of antibodies used: nb169 1∶2; anti-serotonin (Millipore AB938) 1∶500; anti-GFP (Life Technologies A11122) 1∶3000; DAB staining with Vectastain kit (mouse) according to manufacturer’s protocol. Secondary antibodies for immuno-fluorescence: Alexa-488 anti-rabbit (goat) 1∶400 (Life Technologies A11008); Dylight 549 anti-mouse (donkey) 1∶200 (Jackson Laboratories 715-505-151).

## Results and Discussion

Figure 1 depicts maximum intensity z-projections of wild-type brain wholemount preparations stained with 10 different monoclonal antibodies of the Würzburg hybridoma library. Five of the antibodies (ab47, ab135, ab158, ca8, nb139) clearly label cell bodies in the pars intercerebralis, four (ab47, ab158, fb20, nc24) highlight layers of the fan-shaped body, one (ca8) binds to the protocerebral bridge, and all except nb169 detect fine arborisations in tangential layers of the medulla. Each staining pattern is characteristic of the particular antibody and was obtained at least three times (Table 1). Three of the antibodies (ca8, nb168, nb169) also produced reliable stainings in the larval nervous system (Figures 2, 4K-M).

The similarity of mAb nb168 staining (Figure 1G) to the known distribution of pigment dispersing factor (PDF) in *Drosophila* [14], [15] is obvious. Indeed, double stainings of wholemount brains with mAb nb168 and an antiserum against PDH [16] (which cross-reacts with PDF) reveal that the same neurons are labelled by the two antibodies (Figure 3A1-3). Mutation of the *Pdf* gene eliminates mAb nb168 staining and thus demonstrates that mAb nb168 very likely recognizes PDF or its precursor protein (data not shown). Since the hybridoma clones secreting mAb nb168 and mAb nb33, which recognizes PDF precursor protein but not the mature PDF [4], were obtained in the same fusion experiment using B-lymphocytes from a single mouse, it is possible that they derive from a common B cell and thus recognize the same epitope.

The staining pattern of mAb nb169 in the adult brain is of particular interest. It includes one very large cell body in each brain hemisphere and a diffuse network of arborisations, in addition to a small number of cell bodies in the dorso-lateral protocerebrum and the visual system (Figures 1H, 3B2). The distribution of the corresponding antigen was investigated in more detail. Horizontal diamino benzidine (DAB)-stained frozen sections reveal two immuno-positive cell bodies of the lateral protocerebrum with some of their arborisations at the dorso-ventral level of the fan-shaped body (Figure 4A), the two very large cell bodies with their prominent neurite (in an adjacent section) in the posterior protocerebrum at the level of the noduli (Figure 4B), as well as the labelling of cell bodies in the visual system with fine arborisations and varicosities in the accessory medulla, in a tangential layer of the medulla and, conspicuously, in the cell body layer of the lamina (Figure 4C, D). These features were partly reminiscent of stainings using antisera against serotonin [17]. Thus we wanted to determine if the cells identified by mAb nb169 contain serotonin (5HT). Fluorescent double staining with anti-5HT serum and nb169 (Figure 4E, F) clearly demonstrate that mAb nb169 identifies a set of cells similar to, but distinct from, serotonergic neurons in the *Drosophila* brain. The similarity is particularly striking in the visual system where the two antibodies stain distinct neurons of nearly identical axon course and pattern of arborisations in the cellular cortex of the lamina (compare Figure 4C with F, G). This group of cells has been denoted as lamina tangential or wide-field neurons (Lat [18] or Lawf [19]). (Note that the fluorescence of the retina is unspecific). The serotonergic fibers and varicosities in this region have previously been investigated in detail in various insects [20], [21], [22] and are assumed to serve a paracrine function [23]. In the larval ventral ganglion mAb nb169 selectively labels five neurons on each side (Figure 4K-M). Screening patterns of GFP expression driven by subsets of the Janelia Gal4 collection [5], [6] (http://flweb.janelia.org/cgi-bin/flew.cgi) for similarities with our mAb stainings, we discovered two lines (R33G11 and R49C04) that drove GFP expression in two large cell bodies in the caudal protocerebral cellular cortex. Double staining of brains from such flies with mAb nb169 and anti-GFP antiserum revealed that both antibodies label the same two perikarya (Figure 3B1-3 for line R49C04, Figure 4H, enlarged in I1-3 (whole-mount), and 4J1-3 (cryo-section) for line R33G11), while GFP expression was also prominent in various other structures (Figures 3B1, 4H, J2). The co-localization of the nb169 antigen and GFP driven by line R33G11 is also seen in the larval ventral nerve cord (Figure 4M1-3). GFP driven by line R49C04 in larvae, on the other hand, does not co-localize with nb169 antigen. The Gal4 line R33G11 contains a regulatory DNA fragment of the gene CG18405, *Sema-1a*. Gal4 lines driven by other regulatory fragments of this gene show no expression in these cells such that the relation of the antigen recognized by nb169 to the *Sema-1a* gene remains obscure. Obviously, it will be fascinating to learn more about the neurons and the antigen recognized by mAb nb169. The discovery of two Gal4 lines with very different expression patterns overlapping apparently only in the pair of giant neurons labelled by mAb nb169 suggests that it might be possible to generate a line expressing a transgene of choice only in these two giant neurons by split-Gal4 technique [7]. The co-localization results of all tested mAb/Gal4 pairs are summarized in Table 1. Figure 5 shows examples for co-localization of GFP expression driven by Janelia Gal4 lines with stainings by mAb ab47 (Figure 5A-C) and nc24 (Figure 5D-F).

### Strategies towards identifying the antigens for mAbs

Earlier attempts to identify the antigen of mAb nb33 [4] by extensive screening of a cDNA expression library had failed (K. Zinsmaier, unpublished), whereas screening of the same library with mAbs recognizing synaptic proteins was successful [24], [25]. Also, for none of the antibodies described here a reliable Western blot signal from brain homogenate could be obtained, defeating any attempts to identify the antigens by protein purification and mass spectrometry [26], [9]. Thus the classical approaches to the identification of antigens for mAbs cannot be applied. We therefore here present the distribution of the antigens in the *Drosophila* brain and make them available for use in neuroanatomy and for comparison with cell identification by other techniques mentioned above. We first selected a small number of Gal4 lines from the Janelia collection [5], [6] that showed similarity between their expression patterns revealed by GFP and the staining patterns of our mAbs in the adult fly brain. We then investigated by double stainings whether there was any overlap detectable. Table 1 summarizes the results. For mAb nb169 and Gal4-lines R33G11 and R49C04 we find clear co-localization of GFP and antibody binding as described above. Frequently, however, in spite of apparent similarity of staining patterns in the neuropil, e.g. in the fan-shaped body or in horizontal layers of the medulla, the GFP-positive and the mAb-stained cell bodies were distinct albeit often in close proximity. In some cases a small subset of mAb-stained cells also expressed GFP driven by the Gal4-line indicated ((+) in Table 1). The stainings shown in Figure 5 suggest that Gal4 expression from different Gal4 lines may overlap as their GFP reporter is observed in mAb-stained cells at similar positions. However, the positions of cell bodies are somewhat variable from fly to fly such that additional evidence for the overlap will be required.

Since it was shown that mAb nb168 binds to a neuropeptide or its precursor we wanted to test if other antibodies might also identify peptidergic cells. In general, neuropeptides are cleaved from precursor proteins by proteolytic enzymes. Thus many neuropeptide-containing cells can be labelled by GFP driven by 386Y-Gal4, a Gal4 enhancer trap construct under regulatory control of the prohormone convertase 2 gene (*amon*) [8], [27], [28]. Figure 6 shows the co-localization of nb169, ca8, and ab158 antibody staining with amon>GFP expression, supporting a speculation that these three mAbs may perhaps bind to neuropeptide-related antigens.

The interpretation of co-localization (or its lack) of any Gal4-driven GFP expression with a staining pattern obtained with an antibody against an unknown antigen remains difficult. Several mechanisms may be responsible for discrepancies between the Gal4-driven GFP expression patterns and the antigen distributions even if the antigen is the product of the corresponding gene. First, enhancer, silencer, and promoter elements may be widely separated in a regulatory region of a gene and usually combine in a complex, non-additive fashion to produce the final expression pattern. Second, the GFP of the mCD8-GFP construct expressed by Gal4 is targeted to all membranes such that the entire cell is labelled, whereas the antigen recognized by the mAb may be specifically and efficiently targeted to a specific cell compartment and might be detected by the antibody only there. Third, the antigen recognized by the mAb may perhaps be a processed or post-translationally modified version of a gene product and therefore be present only in a subset of the cells expressing the gene. Thus, for each of the mAbs shown here considerable efforts will be necessary in any attempt to identify the antigen. However, in cases where neuronal cell bodies identified by a mAb can be labelled in vivo by GFP in principle it can become feasible to separate these cell bodies and isolate their mRNAs for gene expression profiling [29] or generation of a cell-specific cDNA expression library which can be screened with the mAb. This approach to the identification of unknown antigens labelled by monoclonal antibodies only in a few cells in the brain can now be attempted e.g. with mAb nb169.
