# Correction: Identification and Structural Characterization of Interneurons of the *Drosophila* Brain by Monoclonal Antibodies of the Würzburg Hybridoma Library

**DOI:** 10.1371/annotation/f5b113a4-62bf-41e8-8bdd-fc99271b9d1e

**Published:** 2013-10-01

**Authors:** Beatriz Blanco Redondo, Melanie Bunz, Partho Halder, Madhumala K. Sadanandappa, Barbara Mühlbauer, Felix Erwin, Alois Hofbauer, Veronica Rodrigues, K. VijayRaghavan, Mani Ramaswami, Dirk Rieger, Christian Wegener, Charlotte Förster, Erich Buchner

There was an error in the citation. The correct version is available below.

Blanco Redondo B, Bunz M, Halder P, Sadanandappa MK, Mühlbauer B, et al. (2013) Identification and Structural Characterization of Interneurons of the *Drosophila* Brain by Monoclonal Antibodies of the Würzburg Hybridoma Library. PLoS ONE 8(9): e75420.

doi:10.1371/journal.pone.0075420 

